# Enhanced antineoplastic/therapeutic efficacy using 5-fluorouracil-loaded calcium phosphate nanoparticles

**DOI:** 10.3762/bjnano.9.233

**Published:** 2018-09-20

**Authors:** Shanid Mohiyuddin, Saba Naqvi, Gopinath Packirisamy

**Affiliations:** 1 Department of Biotechnology, Indian Institute of Technology Roorkee, Roorkee, Uttarakhand 247667, India; 2 Nanobiotechnology Laboratory, Centre of Nanotechnology, Indian Institute of Technology Roorkee, Roorkee, Uttarakhand 247667, India

**Keywords:** 5-FU, anticancer drug delivery, apoptosis, calcium phosphate nanoparticles, cell cycle, nanomedicine

## Abstract

In the past few decades, the successful theranostic application of nanomaterials in drug delivery systems has significantly improved the antineoplastic potency of conventional anticancer therapy. Several mechanistic advantages of nanomaterials, such as enhanced permeability, retention, and low toxicity, as well as surface engineering with targeting moieties, can be used as a tool in enhancing the therapeutic efficacy of current approaches. Inorganic calcium phosphate nanoparticles have the potential to increase the therapeutic potential of antiproliferative drugs due to their excellent loading efficiency, biodegradable nature and controlled-release behaviour. Herein, we report a novel system of 5-fluorouracil (5-FU)-loaded calcium phosphate nanoparticles (CaP@5-FU NPs) synthesized via a reverse micelle method. The formation of monodispersed, spherical, crystalline nanoparticles with an approximate diameter of 160–180 nm was confirmed by different methods. The physicochemical characterization of the synthesized CaP@5-FU NPs was done with transmission electron microscopy (TEM), dynamic light scattering (DLS), field emission scanning electron microscopy (FE-SEM), Fourier-transform infrared spectroscopy (FTIR), and X-ray diffraction (XRD). The antineoplastic potential of the CaP@5-FU NPs against colorectal and lung cancer cells was reported. The CaP@5-FU NPs were found to inhibit half the population (IC_50_) of lung adenocarcinoma (A549) cells at 32 μg/mL and colorectal (HCT-15) cancer cells at 48.5 μg/mL treatment. The apoptotic induction of CaP@5-FU NPs was confirmed with acridine orange/ethidium bromide (AO/EB) staining and by examining the morphological changes with Hoechst and rhodamine B staining in a time-dependent manner. The apparent membrane bleb formation was observed in FE-SEM micrographs. The up-regulated proapoptotic and down-regulated antiapoptotic gene expressions were further confirmed with semiquantitative reverse transcriptase polymerase chain reaction (PCR). The increased intracellular reactive oxygen species (ROS) were quantified via flow cytometry upon CaP@5-FU NP treatment. Likewise, the cell cycle analysis was performed to confirm the enhanced apoptotic induction. Our study concludes that the calcium phosphate nanocarriers system, i.e. CaP@5-FU NPs, has higher antineoplastic potential as compared to 5-FU alone and can be used as an improved alternative to the antimitotic drug, which causes severe side effects when administrated alone.

## Introduction

Malignant neoplasms are reported as the second most common cause of mortality around the globe next to cardiovascular disorders. As reported by the World Health Organization, about 7.9 million deaths were documented in 2011 as cancer-related [1]. Furthermore, cancer leads to increased socioeconomic burden to the world. The conventional cancer therapeutic strategies invite unresolved problems in medical prognoses such as nonspecific cytotoxicity, insignificant survival rate, re-initialization of cancer, in vitro evolution of multidrug resistance, and unwanted side effects [2]. The failure of conventional therapeutic strategies indicates that additional efforts should be focused immediately on improving the efficacy. This need could possibly be met by a nanoscale-carrier-facilitated drug delivery system. This rapidly growing field of research has gained interest all over the world with its effective and targeted drug delivery application [3].

Polymer-based nanoformulations have recently shown advancement in drug delivery applications. However, most of the studied biodegradable polymer nanoparticles resulted in acidic or degradation by-products, which may interfere in the drug activity [4] and normal homeostasis of the cell. The degradation of the drug-loaded polymer nanoparticles prior to arriving at the targeted site resulted in serious nonspecific cytotoxicity [4]. Alternative drug delivery strategies using inorganic nanoparticles have also been applied. A magneto-electric nanoparticle (MEN) system was reported as a controlled drug delivery platform for the drug paclitaxel in both in vitro and in vivo studies [5]. Gold nanoparticles conjugated with a trans-activating transcriptional activator (TAT) peptide modification encapsulated with the drug doxorubicin showed enhanced toxicity in brain cancer models [6]. Further, mesoporous silica nanoparticles were successfully used for hepatoma targeted delivery of docetaxel with lactose as the targeting molecule [7]. Curcumin-loaded organically modified silica nanoparticles (ORMOSIL) were studied to check the potential anticancer property of ORMOSIL nanocarriers [8]. However, in some instances, after nanoparticle formation, the nanomaterial was found to be toxic in some metal-based NPs (e.g., gold, silver, iron, and copper), while the bulk counterparts showed less toxicity [9]. The metal ions present at the surface of the particles were found to interact with biomolecules at a comparatively higher rate in nanoformulations compared to the bulk. Thus metal-based NPs applied to biomedical applications could contradict the success of disease prognosis and treatment. Upon synthesis via solvent-extraction or calcination procedures, mesoporous silica nanoparticles (MSNs) were found to inhibit cellular respiration [10] in the in vivo model. Due to the presence of silanols in the calcined MSNs, the aqueous solubility becomes significantly reduced [11], which restricts the clinical application of MSNs.

Calcium phosphate falls into the generally regarded as safe (GRAS) category as outlined by the United States Food and Drug Administration (FDA) [12]. Calcium phosphate cement was previously prepared with embedded anticancer agent 6-mercaptopurine (6-MP) showed a sustained and slow release profile [13]. In a normal metabolism, the concentration of calcium phosphate present in the bloodstream was estimated to be 1–5 mM [14]. This is the biological tolerance limit for the use of calcium phosphate as an effective carrier for therapeutic strategies in real time [15] where other carriers failed in clinical trials entirely. Furthermore, the localized and controlled delivery through various routes, cost-effective preparation, high bioavailability, increased chemical stability, stimuli-responsive behaviour, pH-dependent properties, surface engineering characteristics, low or no antigenicity [16] and aqueous solubility of calcium phosphate nanoparticles greatly enhances the therapeutic efficacy. The exploitation of calcium phosphate nanoparticles in biomedical applications is extended to tissue engineering, gene/siRNA delivery, anticancer drug delivery, protein and antigen delivery, vaccine delivery, insulin as well as imaging probe or contrasting agent delivery for bio-imaging.

5-Fluorouracil (5-FU), a well-known anticancer agent introduced in 1958, displays higher therapeutic efficacy in solid tumours like colon, rectum and breast cancers. 5-FU exhibits increased bioavailability and a versatile generation of antineoplastic properties and is a commonly administered chemotherapeutic drug. Due to its nucleotide analogue chemical structure, 5-FU enables the misincorporation of the fluoro-nucleotides into RNA and DNA and effectively inhibits synthetic enzyme (i.e., thymidylate) synthase, which further results in the arrest of cellular growth and proliferation. However, due to the increased rate of metabolism in the blood, 5-FU has a short biological half-life (8–20 min). In addition, it has shown nonspecific toxicity in healthy cells. Also, rapid renal clearance, quick liver-mediated metabolism by dihydro-pyrimidine dehydrogenase (DPD) enzyme, and increased digestive distress restricts the use of 5-FU in cancer therapy. It was estimated that about 80% of administered 5-FU metabolizes in the liver and kidney and is detoxified and excreted as F-β-alanine through urine [17].

The 5-FU-loaded nanoparticles employed in anticancer therapy have been gaining research interest. Recently, selenium nanoparticles were surface-functionalized with 5-FU and resulted in enhanced cellular uptake and efficacy [18]. Furthermore, a chitosan/gold nanocomposite was employed as a load carrier for 5-FU with an encapsulation efficiency of 96% [19]. Interestingly, monomeric self-assembled nucleoside nanoparticles (SNNPs) loaded with 5-FU were shown to inhibit the cancer cells of an oral squamous cell carcinoma (OSCC) mouse xenograft model with increased blood retention time [20]. Furthermore, a poly(amidoamine) (PAMAM) dendrimer stabilized with a silver nanoparticle surface for the encapsulation of 5-FU showed synergistic growth inhibition in A549 and MCF-7 cells [21].

Herein we report the synthesis of calcium phosphate nanoparticles loaded with 5-FU (CaP@5-FU NPs) with the goal of demonstrating enhanced efficacy in lung and colorectal cancer treatment in cell lines. The simple and reproducible reverse micellar microemulsion method was employed for the synthesis of CaP@5-FU NPs.

## Results and Discussion

### Synthesis of CaP@5-FU NPs

**Figure 1:**
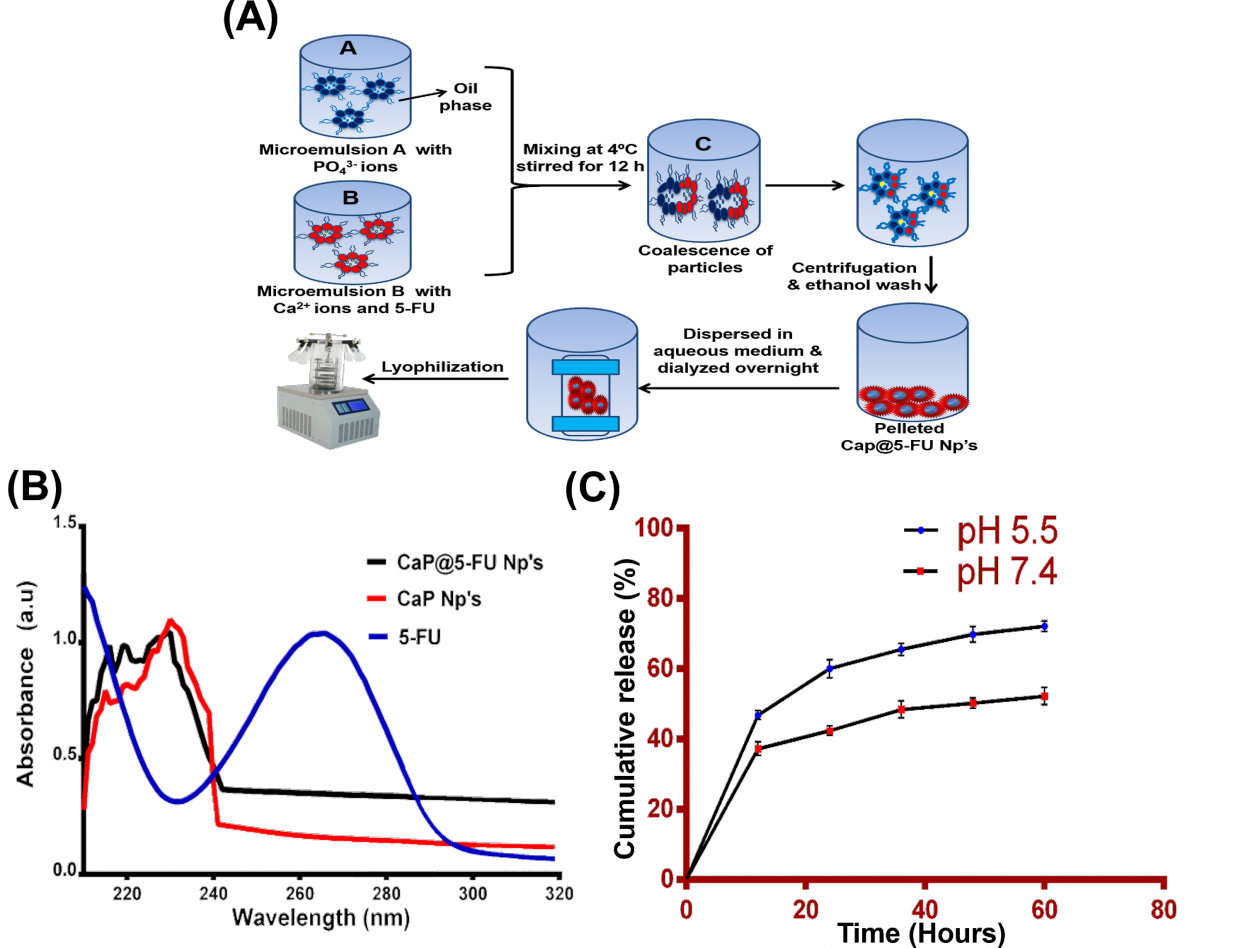
(A) Schematic representation for the preparation of CaP@5-FU NPs. (B) The UV–visible spectrum of CaP@5-FU NPs (black line), CaP NPs (red line) and 5-FU (blue line). (C) The pH-triggered release profile of 5-FU from the CaP@5-FU NPs with respect to time.

The microemulsion reverse micelle method [12] was adopted for the effective synthesis of calcium phosphate nanoparticles. Furthermore, in situ loading of the anticancer drug (5-FU) during the synthesis yielded efficient loading of 5-FU inside the aqueous core, as depicted in Figure 1A. During self-assembly, the aqueous soluble drug (5-FU) along with the CaP precursors formed spheroidal aggregates known as a reverse micellar assembly [22]. The size of the particles can be effectively controlled by altering the water–surfactant molar ratio (*M*_W_). By restricting the water–surfactant molar ratio up to 10 (i.e., *M*_W_ < 10) the reverse micelles can be readily formed with narrow size distribution in a synthetic reaction system [23]. The water molecules restricted inside hydrophilic core resulted in swollen micelles that were uniformly dispersed in oil, forming a water-in-oil (W/O) microemulsion system [24]. Highly monodisperse, stable, size controllable and chemically reproducible nanoparticles can be formed by adopting the aforementioned method. The anionic surfactant (AOT) in the organic solvent forms the oil dispersion media, while aqueous-dissolved 5-FU comprises a dispersed phase. The calculated drug loading efficiency of 5-FU in CaP nanoparticles was 64%, which represents an improved drug encapsulation efficiency. The final yield of the chemical synthesis was found to be 10 mg/100 mL.

### UV–visible spectroscopic studies

The synthesized CaP@5-FU NPs were characterized with UV–vis spectroscopy over a range of 200 to 800 nm. The spectrum obtained displayed a characteristic peak at 230 nm [25] for unloaded CaP NPs and CaP@5-FU NPs as shown in Figure 1B. Also an absorption maximum was found at 265 nm for 5-FU, which was depressed in the drug-loaded nanoparticles (CaP@5-FU). This might be due to fact that the drug was loaded in the aqueous core of the nanoparticles [26]. Furthermore, upon drug loading, insignificant changes were found in the absorption spectrum of the nanoparticles.

### pH-triggered drug release study

To understand the rate of 5-FU release from CaP@5-FU NPs, we employed the dialysis bag method. An acidic (in vitro model for the tumour microenvironment) condition correlates with drug release in the acidic tumour regime. On the other hand, physiological conditions at pH 7.4 represent the healthy cell environment in the study. Cancer cells flourish in an acidic microenvironment, which enhances the tumour progression by evading the normal immune response and metastasis development. The secretion of lactate in the anaerobic glycolysis (with CO_2_ as a by-product of the metabolism) hypoxia environment results in the acidic microenvironment [25].

In our study, we demonstrated a biphasic release of 5-FU from CaP@5-FU NPs with the characteristic initial burst release followed by slow and sustained release [27]. Within 12 hours, only 36% drug release in physiological pH was shown, whereas 47% was released in acidic pH conditions (Figure 1C). At the end of the study (i.e., 60 h), the release was 72% and 52% in acidic and physiological pH, respectively. This experiment demonstrates that the drug 5-FU can be effectively released into a tumour microenvironment from CaP@5-FU NPs, rather than into healthy cells. The enhanced accumulation of the drug in acidic pH correlates with the characteristic release profile in the tumour microenvironment [28]. This can be explained by the hydrogen-bonding interaction between the drug and nanoparticles, which might be strong in physiological pH (pH 7.4) and exhibits insignificant drug release. On the other hand, with increased acidic conditions, there is an augmented probability of more H^+^ ions available to counteract the nanoparticle–drug formulation, thereby reducing the interactions [28]. This property of the proposed nanoparticle drug delivery system significantly improves the efficacy of the treatment in real time and reduces the nonspecific toxicity.

### Particle size determination by dynamic light scattering

**Figure 2:**
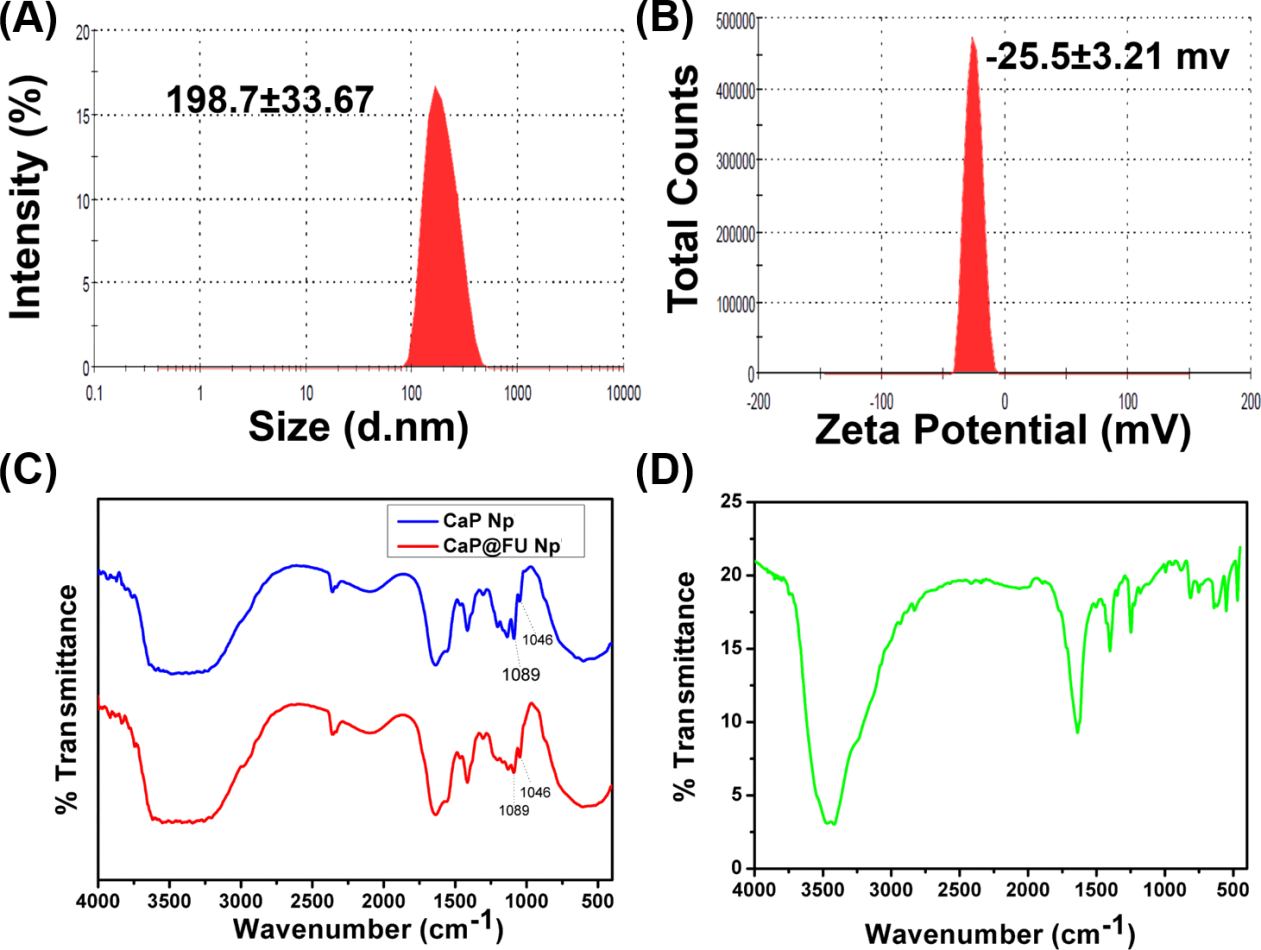
(A) The hydrodynamic diameter and (B) zeta potential of CaP@5-FU NPs in PBS buffer. (C) The FTIR spectra of CaP NPs (blue line) and CaP@5-FU NPs (red line) along with (D) 5-FU.

The diameter of the synthesized CaP@5-FU NPs was analysed with dynamic light scattering and a mean hydrodynamic diameter of 198 ± 33.67 nm was determined (Figure 2A). The particles showed less polydispersity (i.e., a lower polydispersity index (PDI), 0.112) in the aqueous medium and high colloidal stability. The size-controllable synthetic procedure offers enhanced therapeutic efficacy in real time. The size of the aqueous dispersed particles was confirmed by TEM and SEM analysis.

### Zeta potential analysis

The magnitude of the surface charge of the synthesized CaP@5-FU NPs was characterized by zeta potential analysis. The increased electrostatic repulsion between particles corresponding to a higher magnitude of the zeta potential results in reduced agglomeration and enhanced colloidal stability [29]. Since the particle stability mainly depends on the electrical charge of the surface, properties such as cellular uptake, rate of drug release, and blood retention time are directly correlated with the zeta potential value. Furthermore, the zeta potential value of calcium phosphate nanoparticles was found to have a direct association with Ca/P molar ratio [30]. The molar ratio of the formed nanoparticles in the current study was 3.88.

The synthesized CaP@5-FU NPs were found to have a zeta potential of −25.5 mV (Figure 2B). This confirms the nanoparticle formation as previous reports of the zeta potential for calcium phosphate nanoparticles were −23 mV [31]. The colloidal stability of synthesized CaP@5-FU NPs was monitored for one month in regular intervals and no significant change in the zeta potential value was observed, confirming the stability of the colloid (data not shown).

### Fourier-transform infrared spectroscopy analysis

The vibrational spectrum in the infrared region of synthesized CaP@5-FU NPs and CaP NPs with the drug 5-FU was observed to establish the chemical confirmation of the synthesized material with respect to the functional group. The infrared electromagnetic radiation induced dipole moment of the molecules is used to provide information on the functional group. We demonstrated that phosphate ester (with a signature peak at 1300–1240 cm^−1^) having a characteristic P=O stretch was found in the both CaP@5-FU NPs and CaP NPs with drug loading (Figure 2C). The characteristic FTIR peaks were found in both unloaded and drug-loaded nanoparticles, which was confirmed by the PO_4_^3−^ peaks at 1089 and 1046 cm^−1^ and corresponds to previous reports [32]. The calcium phosphate nanoparticles exhibited FTIR peaks in the range of 1000–1100 cm^−1^, corresponding to the phosphate bending. Evidence of P=O bonds was confirmed by a peak at 1200–1100 cm^−1^ [32] in the two nanoparticle samples synthesized in addition to P–O bonds in the range of 1100–1000 cm^−1^. In addition, the FTIR spectrum of 5-FU alone was collected for comparative analysis as shown in Figure 2D.

### X-ray diffraction pattern

**Figure 3:**
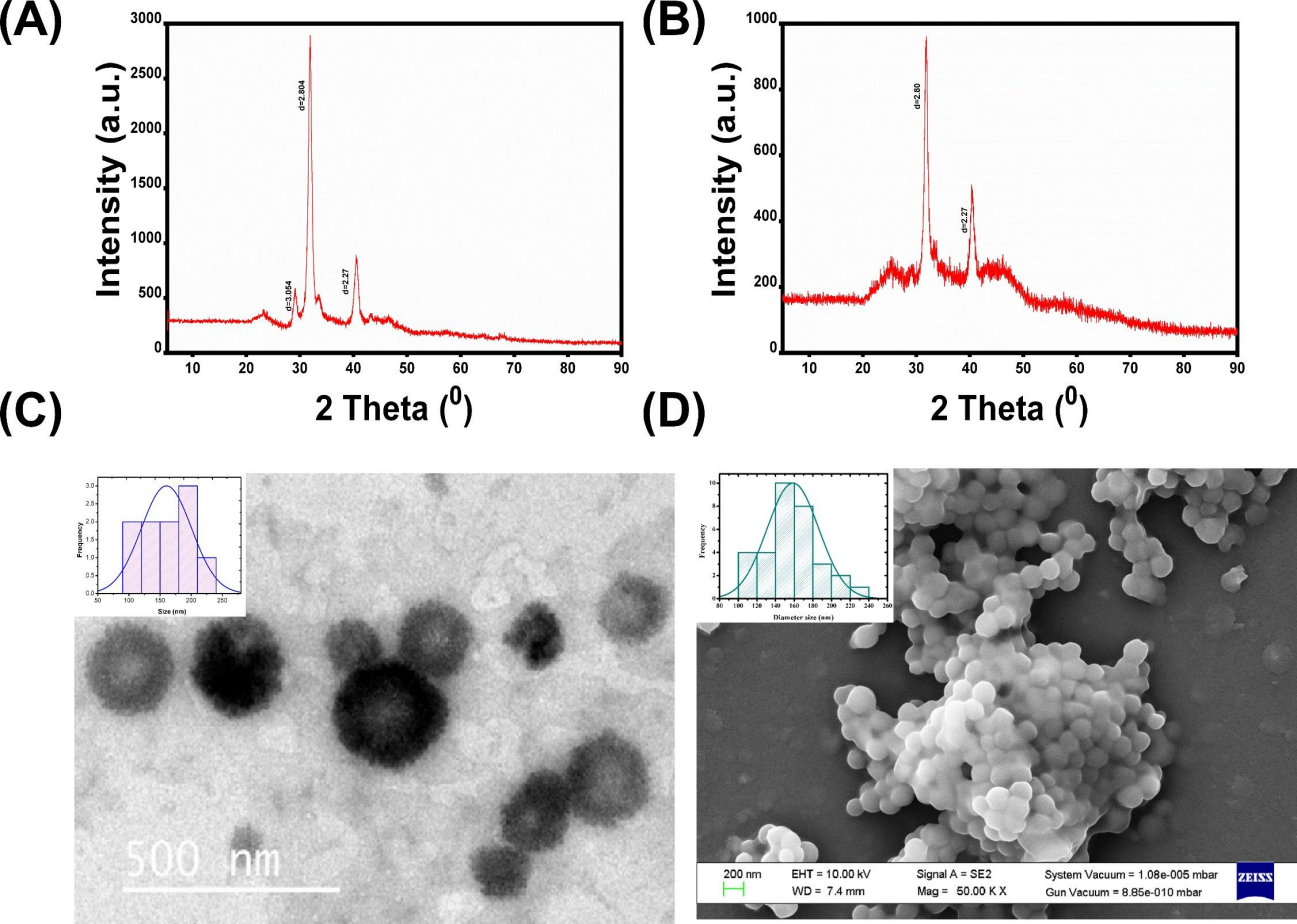
The X-ray diffraction pattern of (A) CaP NPs and (B) CaP@5-FU NPs. (C) TEM and (D) FE-SEM micrographs of CaP@5-FU NPs.

X-ray diffraction (XRD) analysis was performed to assess the crystallinity of both CaP@5-FU NPs and CaP NPs, as shown in Figure 3B and Figure 3A, respectively. A diffraction pattern with a 2θ value of 31.8 (*d* value 2.804) confirms the hydroxyapatite (HAP) formation [33]. The width of the peak confers the nanocrystalline nature [34] of the particle. The crystalline peak of HAP (Ca_10_(PO_4_)_6_(OH)_2_) at 2θ = 31.8 was reduced upon drug loading. From this information, it can be deduced that HAP (JCPDS card number 9–0432), the most stable form of calcium phosphate, was found to be the integral constituent of the both CaP NPs and CaP@5-FU NPs.

### Transmission electron microscopy analysis

The surface morphology and internal composition of CaP@5-FU NPs were observed by transmission electron microscopic (TEM) images. The NPs were found to have a mean diameter of 161 ± 29.65 nm, as shown in the Figure 3C. The spherical structure can be readily observed in the negatively stained grids. The mean diameter as obtained from TEM micrographs is smaller than the hydrodynamic diameter concluded from the DLS analysis (Figure 2A) due to the lack of hydration layer [35] (dry sample) in TEM. On the other hand, the DLS analysis will determine the size of the particle in the solution.

### Scanning electron microscopy studies

FE-SEM was performed to confirm the particle size, morphology, and elemental characterization. The synthesized CaP@5-FU NPs were found to have a mean diameter of 158 ± 27.6 nm. The spherical shape [36] of the CaP@5-FU NPs (Figure 3D) confirmed that the nanoparticles were properly synthesized via the reverse micellar method. The presence of calcium, oxygen, phosphorous was confirmed by energy-dispersive X-ray spectroscopy (EDS) (Figure S2, Supporting Information File 1). The absence of fluorine in the EDS analysis confirms the presence of the drug encapsulated inside the aqueous core of the nanoparticles. The size of the as-synthesized CaP@5-FU NPs was thus confirmed by FE-SEM. The morphological characterization was also confirmed with the help of atomic force microscopic (AFM) images (Figure S1, Supporting Information File 1). The size of the nanoparticles was approximated as 163 ± 24.12 nm.

### MTT assay

**Figure 4:**
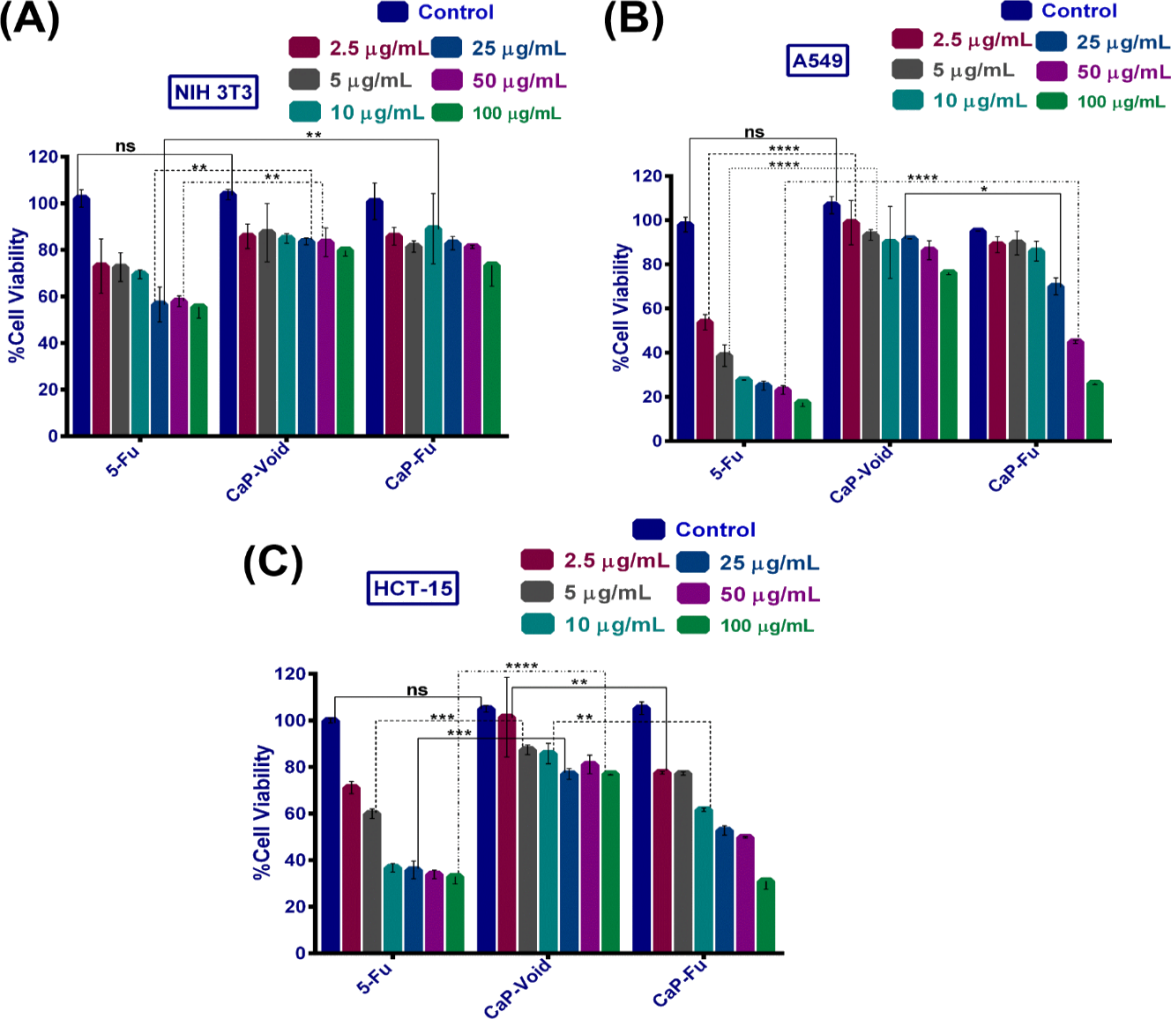
Analysis of cell viability by MTT assay for (A) NIH 3T3, (B) A549 and (C) HCT-15 cells upon CaP@5-FU NP, CaP NP and 5-FU treatment for 24 h. Two-way analysis of variance (ANOVA) followed by Tukey’s multiple comparison test were performed for the statistical significance assessment between the mean of the group (*p < 0.05, **p < 0.005, ***p < 0.001, ns – non significant).

To determine the antineoplastic potential of 5-FU, CaP@5-FU NPs and CaP NPs, we performed an MTT assay in Duke’s type C colorectal adenocarcinoma (HCT-15) and lung adenocarcinoma (A549), which are considered to be the in vitro cancer model. This was followed by mouse fibroblast cell line (NIH 3T3) and human embryonic kidney cell line (HEK-293) as normal cell line models. From the assay, we observed 22.9% of the cells were inhibited at a higher concentration (100 µg/mL) of CaP NPs in HCT-15 cell lines (Figure 4C). The same was found in 22.3% and 18.68% of inhibition in A549 and NIH 3T3 cell lines as demonstrated in Figure 4B and Figure 4A, respectively. The comparative low cytotoxicity of CaP NPs as a carrier (even at a higher concentration of 100 µg/mL) reveals the suitability of this material as a carrier in a successful drug delivery platform. The elevated ionic concentration of Ca^2+^ in the cytoplasm leads to some biochemical mechanism triggers for cell death via either necrotic or apoptotic processes. Some reports suggest that Ca^2+^ can produce a detrimental effect in pathological and toxicological aspects [37]. A misbalance in the intracellular Ca^2+^ homeostasis results in the progression of cell death. The ≈20% growth inhibition due to CaP NPs might be due to hypercalcemia [38] or other cellular interference issues caused by calcium.

The cytotoxic examination was further extended using free drug (no NPs) and the CaP@5-FU NPs sample at the same concentration and time as previously mentioned. In the free drug test (i.e., 5-FU), we observed a great reduction in cell viability in both cancer models (HCT-15 and A549 cells), whereas it was less effective in the normal cell line (NIH 3T3). We observed an IC_50_ value of 4.5 µg/mL and 6.5 µg/mL in A549 and HCT-15 cell lines, respectively (Figure 4B,C). Furthermore, upon administration of 5-FU at a concentration of 100 µg/mL, a 67.02% and 81.5% reduction in cell growth in HCT-15 and A549 cell lines was observed. CaP@5-FU NPs showed a concentration-dependent toxicity on the cells irrespective of the cell lines used. In NIH 3T3 cells, considerably low toxicity was found (Figure 4A) with respect to cancer cells. The increased metabolism in cancer cells might be the reason for the enhanced cellular uptake [39] of the particles and thus the enhanced toxicity in the tumour cells. The sustained release of 5-FU from the CaP@5-FU NPs, as described in the pH-triggered release study, was found to correlate with the findings of the MTT assay. The HCT-15 cell lines showed a saturation limit of the free drug after 10 µg/mL, whereas treatment with CaP@5-FU NPs does not show much effect on the same cells. Moreover, free 5-FU at a concentration of 100 µg/mL revealed a 32.98% viability in HCT-15 cells. However, CaP@5-FU NPs showed a viability of 31.01% at this higher concentration, thus indicating better efficacy as compared to the free drug. This might be due to the mechanistic action of p-glycoprotein-mediated discharging [40] of the drug at the onset of the saturation concentration. To substantiate the biocompatibility of the nanoparticles, we extended the MTT assay to HEK-293 cells. The results obtained in this study indicate that 5-FU has an IC_50_ of 4.2 µg/mL, whereas incubation at an increased concentration of 100 µg/mL resulted in 17.44% survival of cells (Figure S3, Supporting Information File 1). Unloaded CaP NPs resulted in a 76.24% cell viability at 100 µg/mL treatment. Conversely, CaP@5-FU NPs showed a survival of 26.22% of cells after 100 µg/mL treatment. These results enable us to confirm drug-loaded-NP-induced toxicity on the cells (for all cell lines used) and to exclude carrier-mediated toxicity.

### Acridine orange/ethidium bromide (AO/EB) assay

**Figure 5:**
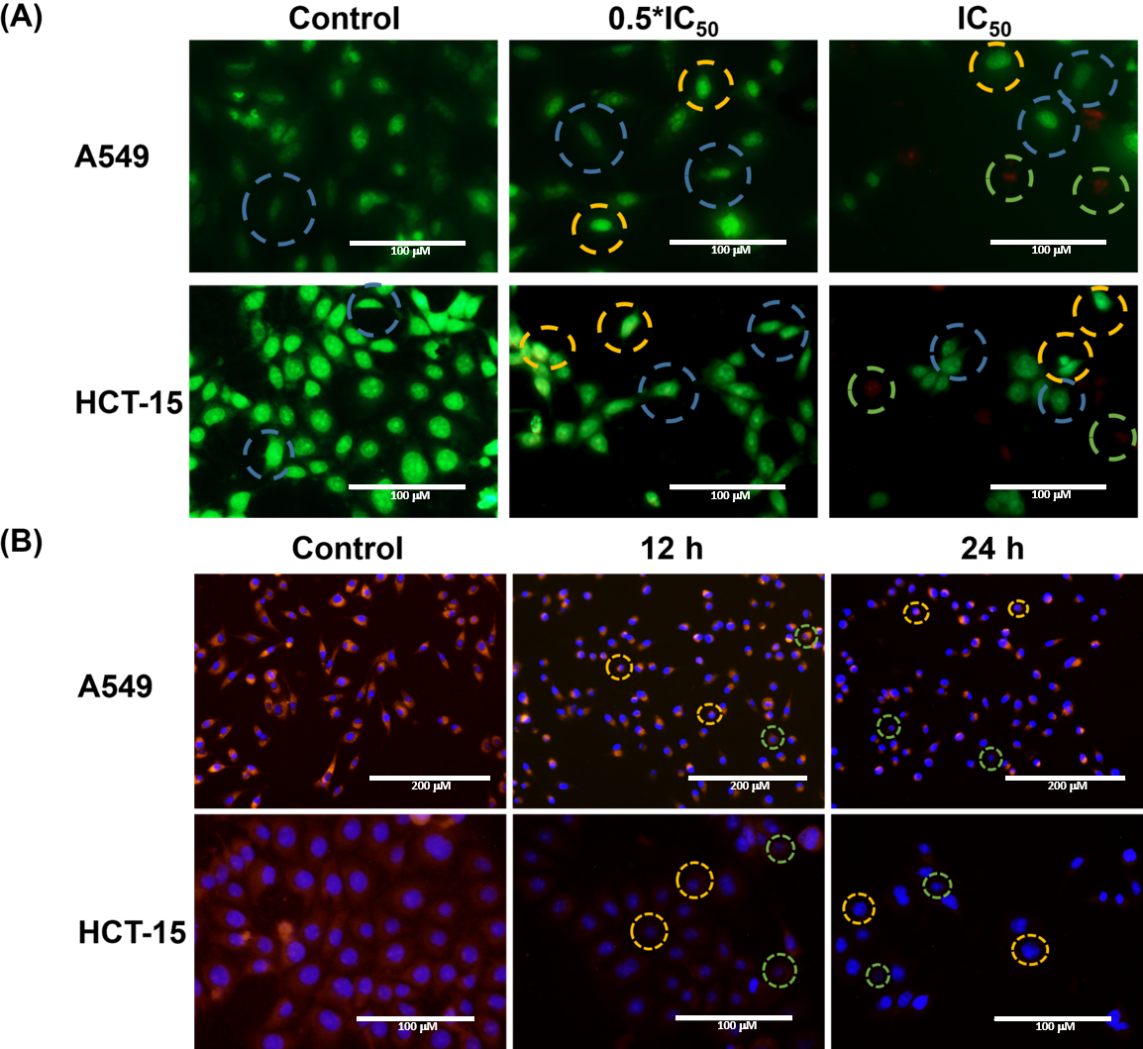
Fluorescence microscopy images of (A) AO/EB stained A549 and HCT-15 cells after 0.5 IC_50_ and IC_50_ with CaP@5-FU NP treatment for 24 h, showing multiple apoptotic events and (B) Hoechst 33342 and rhodamine B stained A549 and HCT-15 cells upon 12 h and 24 h incubation with CaP@5-FU NPs with respect to the untreated control visualises the morphological changes after CaP@5-FU NP treatment. Scale bars = 100 µm, except for A549 with scale bars = 200 µm.

The mechanistic evolution of 5-FU-induced apoptosis was confirmed with dual staining of acridine orange/ethidium bromide (AO/EB) in the cells. The differential staining of the combination AO/EB reveals the different apoptotic phases. AO, with a fluorescence excitation/emission of 431/520 nm, can permeate the cells and yields a bright green fluorescence upon excitation. Likewise, EB, having an excitation/emission of 524/605 nm, provides an orange fluorescence upon intercalculation with dsDNA and can permeate into membrane-compromised cells only. The degree of EB staining in the cells dictates the proportion of the cell membrane compromised. During the early apoptotic stages, less EB is taken up by the cells, followed intermixed red–green fluorescence emission [41]. Like the MTT assay result, the 0.5×IC_50_ of CaP@5-FU NPs treated cells showed early apoptotic phase with less EB fluorescence in both the HCT-15 and A549 cancer cell lines. In the Figure 5A, the blue dotted circle indicates a viable cell, yellow are early apoptotic cells and green shows late apoptotic cells, which were found in the 0.5×IC_50_ and IC_50_ study of CaP@5-FU NP treated cells. The proportional reduction in the cell number count was observed in both the cell lines when compared to the untreated control. The green fluorescence from AO found in healthy cells (untreated control) was reduced in the case of NP-incubated cells, as inferred by the membrane that was compromised after particle incubation. The thymidylate synthase inhibition of 5-FU in the apoptotic induction of cells was qualitatively determined by the AO/EB assay. Furthermore, more evidence of late apoptotic stages was observed in IC_50_ treated cells (Figure 5A). A significant increase of EB fluorescence confirms the enhanced apoptotic induction in the cells and the total cell count was found to be drastically reduced. With this assay, drug-induced apoptosis was confirmed by CaP@5-FU NP treatment.

### Hoechst 33342 and rhodamine B

Apoptotic cells show specific characteristic features such as morphological changes, nuclear fragmentation, and cytoplasmic constriction [41]. The nucleic acid specific dye Hoechst 33342 and the cytoplasmic dye rhodamine B were used to stain the cells treated with an IC_50_ concentration of CaP@5-FU NPs incubated for different time periods. The morphological changes can be effectively tracked by microscopic examination after the staining procedure.

Hoechst 33342 is a fluorescent DNA intercalating dye, which stains apoptotic cells or nuclei and yields blue florescence when bound to dsDNA in the cell nucleus [42] with excitation/emission wavelength at 352/455 nm. Rhodamine B, with an excitation/emission wavelength of 543/565 nm, provides a red fluorescence emission. A drastic decrease in the Hoechst 33342 dye signal denotes the nuclear fragmentation leaving the blue-dyed nucleus, whereas reduction in the rhodamine B dye signal infers cytoplasmic constriction. In this assay, both the untreated A549 and HCT-15 cells display bright red and blue fluorescence, indicating healthy, intact cells (Figure 5B). After a treatment with IC_50_ concentration of CaP@5-FU NPs, the cells showed significant cytoplasmic constriction (yellow dotted circle) and chromatin condensation (green dotted circle) irrespective of incubation time. Considerable changes to the morphology were observed when the incubation time was increased. This allows us to hypothesise that the concentration (obtained from AO/EB results) and exposure time play a crucial role in the antineoplastic evolution of CaP@5-FU NPs. The morphological visualization of the HCT-15 and HEK 293 cells after incubation with CaP@5-FU NPs was performed by bright-field microscopy and demonstrated considerable morphological changes as depicted in Figure S4, Supporting Information File 1.

### Membrane blebbing determination by FE-SEM

**Figure 6:**
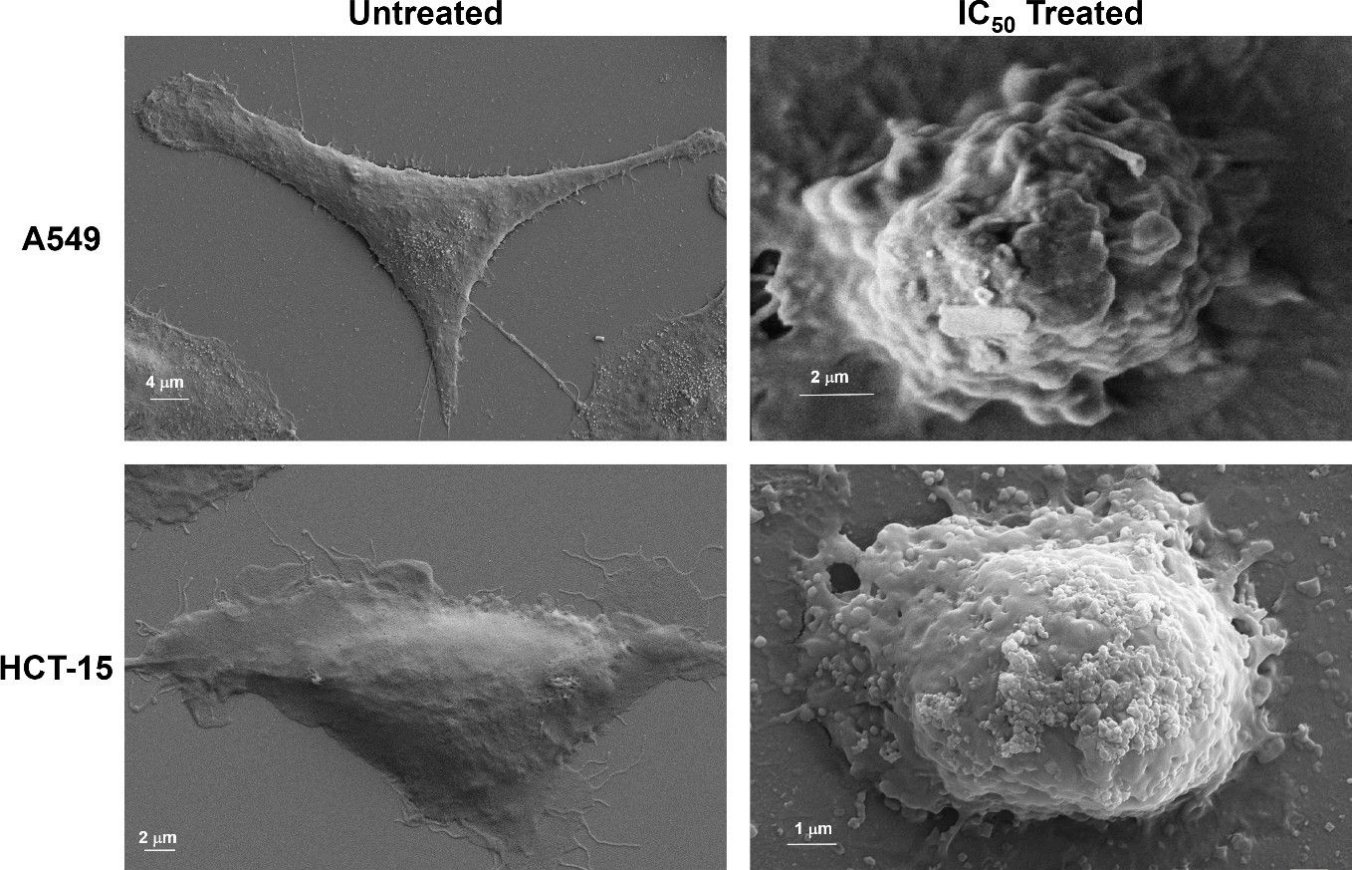
FE-SEM micrograph of untreated control cells A549 and HCT-15 along with CaP@5-FU treated cells at IC_50_ concentration.

As the cell progress into the apoptosis phase, the distinct formation of bleb on the plasma membrane is the most obvious transformational aspect of apoptosis. Membrane blebbing in cell membrane is evidence that the cell is undergoing apoptosis along with cytoplasmic contraction and DNA fragmentation. To visualize the morphological changes occurring during membrane blebbing, we conducted FE-SEM microscopy to obtain high-resolution micrographs of CaP@5-FU NP treated cells. In the induction of apoptotsis in cells, an activated ROCK1 protein is involved in the cellular shrinking and formation of membrane blebbing. The mechanistic action of activated ROCK1 to stabilize the actin, phosphorylate myosin and finally the coupling of the actin-myosin cytoskeleton to the plasma membrane, eventually leads to cell contraction and bleb formation on the cell membrane [43]. In our study, a well-structured cytoskeleton was visualised in an untreated control of both A549 and HCT-15 (Figure 6) cells, showing healthy cells. The active attachment on the surface is apparent from the cytoskeleton and was detected in the untreated control. Upon CaP@5-FU NP treatment, the cells start to shrink (Figure 6) due to the mechanistic evolution of 5-FU-induced apoptosis. The contraction of cells can be quantified by the scale bar measurement. The formation of clear bleb on the membrane can also be confirmed from the micrograph. Even the outer cytoskeleton of treated cells was found to be non-intact and structureless. This assay further confirms the apoptotic induction by the CaP@5-FU NPs in both lung and colorectal cancer cells in vitro*.*

### Intracellular reactive oxygen species quantification via flow cytometry

**Figure 7:**
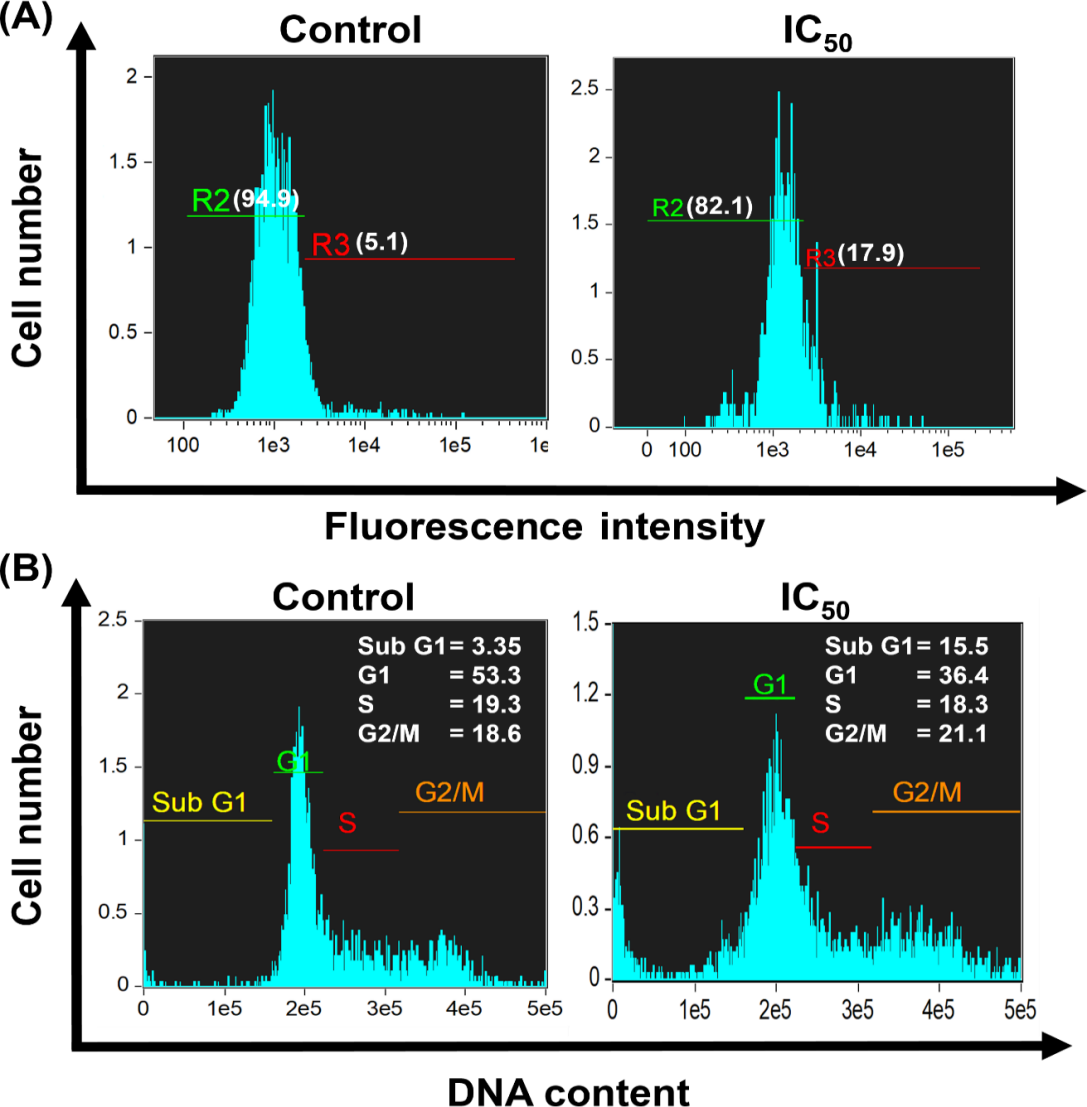
Flow cytometric analysis of (A) CellRox deep red for ROS quantification and intensity of propidium iodide (PI) fluorescence (B) for cell cycle analysis in IC_50_ CaP@5-FU treated HCT-15 cells with respect to untreated control.

**Figure 8:**
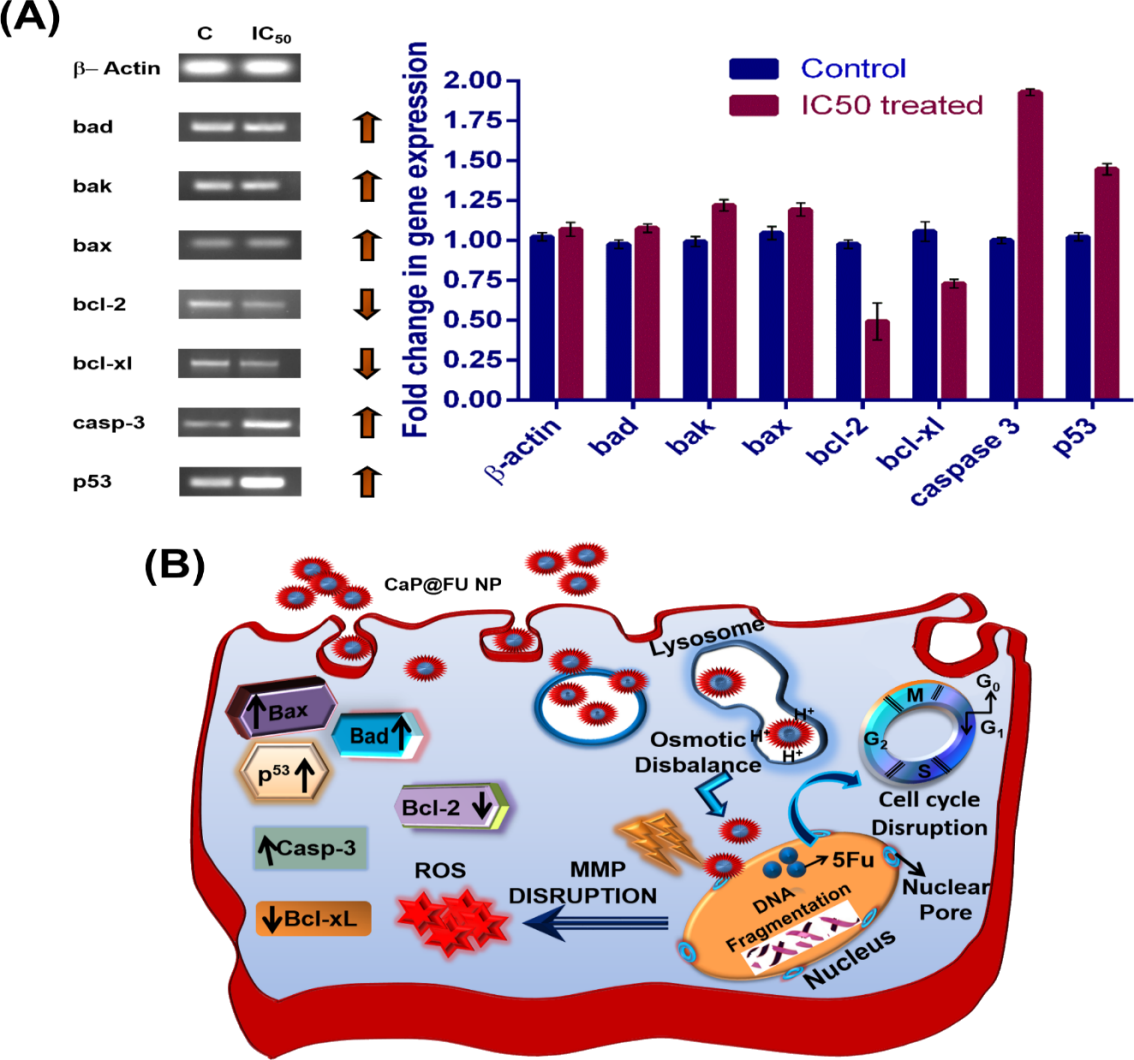
(A) Gene expression studies of proapoptotic and antiapoptotic genes in untreated and CaP@5-FU NP-treated HCT-15 cells using semiquantitative reverse transcriptase PCR method. (B) Schematic representation of a proposed mechanism for CaP@5-FU-induced apoptosis in cells.

The apoptotic induction was experimentally determined by staining techniques, as well as fluorescence and electron microscopy. We further investigated the probable cause of apoptotic induction in cells by CaP@5-FU NPs. It was reported that 5-FU-mediated cell death involves the generation of intracellular reactive oxygen species (ROS) molecules (e.g., O_2_^−^, OH^·^, H_2_O_2_) [44]. The elevated level of ROS species interferes with the normal metabolism of the cells by disrupting cell structures such as lipids, proteins and DNA [45]. This increased oxidative stress later results in cellular damage and apoptosis. To substantiate the ROS generation upon CaP@5-FU NP incubation, we performed a flow cytometric assay with CellROX deep red staining in the HCT-15 cell line. The cell-permeable CellROX deep red dye has an excitation/emission wavelength at 644/665 nm. In its reduced state, CellROX deep red has no or little fluorescence. Upon oxidation by reactive oxygen species, the increased bright red fluorescence emission of CellROX deep red dye can be easily quantified by fluorescence via flow cytometry [46]. The finding was quite interesting whereby 5.1% of the population of the untreated control (Figure 7A) showed ROS-influenced cells, whereas the IC_50_ concentration of CaP@5-FU NPs showed a 17.9% ROS-influenced cell population. The ROS-induction in the untreated control might be due to the normal cellular metabolic by-product due to ROS. This results in an approximate 3.5-times increase in the ROS generation for cells treated with CaP@5-FU NPs. The results thus suggested the antineoplastic potential of CaP@5-FU NPs by inducing intracellular ROS. These results provide us with insight into our proposed mechanism (see below Figure 8B).

### Cell cycle analysis

With the understanding of apoptotic induction occuring in the CaP@5-FU NP-treated HCT-15 cells, we further investigated the effect of CaP@5-FU NPs on the cell cycle. The influence of CaP@5-FU NPs on the cellular metabolism was taken into consideration with cell cycle analysis. We performed propidium iodide staining for DNA quantification and to unveil the different cell cycle phases. Propidium iodide (PI), a fluorogenic dye, intercalates with nucleic acid in a stoichiometric fashion and can be used as an effective indicator dye in cellular DNA quantification with flow cytometry [47]. In apoptotic cells, a broad sub-G1 peak was observed where the cells underwent a hypodiploid nuclear transformation. On the contrary, a narrow sub-G1 peak in the control cells was observed due to normal (diploid) DNA [48]. In the process of apoptosis, nuclear fragmentation is the key step to identify the apoptotic cells. Furthermore, the loss of DNA during apoptotic body formation resulted in reduced DNA content [49], which results in less binding of PI to the DNA. With the DNA content histogram (obtained indirectly by PI fluorescence), we can understand the cell cycle phases (sub-G1, G0/G1, S and G2M phases) and detect changes at the subcellular level during the course of incubation. In the flow cytometry analysis, we observed a 3.35% count of the sub-G1 population in the untreated control and 15.5% IC_50_ of CaP@5-FU NP-treated cells (Figure 7B). An increase of 4.63-times in the apoptotic-induced cell population corroborates the previous results of the ROS induction assay, AO/EB observations, and SEM micrographs. The results infer the detrimental effect of 5-FU-induced toxicity on cell growth and multiplication [50]. The change in the mitochondrial membrane potential over the course of a 24 h treatment with CaP@5-FU NPs in HCT-15 cells was validated (Figure S5, Supporting Information File 1) by using JC-9 dye as the mitochondrial specific dye. The CaP@5-FU NP treatment demonstrated a 2.37-times increase in cell population with loss in mitochondrial membrane potential.

### Semiquantitative reverse transcription polymerase chain reaction assay for gene expression studies

The apoptotic induction capability of CaP@5-FU NPs in HCT-15 cells was further validated by gene expression. For this, a semiquantitative polymerase chain reaction (PCR) analysis of antiapoptotic genes and proapoptotic genes in CaP@5-FU NP-treated HCT-15 cells was undertaken. The gene-specific amplification in a thermal cycler enables the quantification of protein expression. The apoptosis can occur through either the intrinsic mitochondrial-mediated pathway or by the extrinsic death receptor (FasL) facilitated pathway. With the incubation followed by cellular uptake of CaP@5-FU NPs, the 5-FU released in the cytosol results in thymidylate synthase inhibition. This is eventually followed by signal cascade for the intracellular mitochondrial-mediated apoptotic signalling pathway. The proapoptotic genes, including p53 (tumour suppressor protein), BAX (bcl-2-associated X protein), BAK (bcl-2 homologous antagonist killer), caspase-3 and BAD (bcl-2-associated death promoter) were amplified in a thermal cycler. On the other hand, the antiapoptotic genes Bcl-2 (B-cell lymphoma 2) and Bcl-xL (B-cell lymphoma-extra-large) [51] along with β-actin as an internal control were employed for PCR amplification with gene-specific primer as described in Table S1, Supporting Information File 1. This result demonstrated the up-regulated expression of proapoptotic genes and down-regulated antiapoptotic genes, which clearly suggests the apoptotic induction upon CaP@5-FU NP incubation occurred inside the cells. The 5-FU-mediated inhibition of the thymidylate synthase causes the release of cytochrome c from mitochondria through the mitochondrial membrane channel by the interaction of BAX and BAK proteins. Located on the outer mitochondrial membrane, Bcl-2 and Bcl-xL inhibit the actions of BAX and BAK, thereby reducing the cytochrome c flow. The decreased expression resulted in less inhibition of BAK and BAX. Also, BAD binds with Bcl-2 and Bcl-xL to form a heterodimer, thereby inactivating Bcl-2 and Bcl-xL, allowing BAX and BAK to be involved in apoptosis. The up-regulated protein expression of BAK, BAX and BAD (proapoptotic genes) and down-regulated protein expression of Bcl-2 and Bcl-xL (antiapoptotic genes) were found in the treated HCT-15 cells (Figure 8A), which enabled the easy translocation of cytochrome c to the cytosol. P53 plays a pivotal role in homeostatic regulation, growth and response in oxidative stress conditions in order to control or repair diseased cells by initiating apoptosis. As expected, the elevated expression of p53 up to 1.44-fold (Figure 8A) clearly suggests that apoptotic induction occurred in the treated cells. Furthermore, caspase-3 is an important protein involved in the cascade of events and results in the transformation of pro-caspase into active caspase. The expression level of caspase-3 was found to be 1.93-times higher than the untreated control, which confirmed the apoptotic induction after CaP@5-FU NP incubation. The elevated expression of p53, caspase-3, BAK, BAD and BAX, along with the diminished expression of Bcl-2 and Bcl-xL, correlates with the previously reported apoptotic signalling pathways [52].

## Conclusion

Aqueous, dispersible, 5-FU-encapsulated calcium phosphate nanoparticles were prepared by a reverse micelle microemulsion method. The highly monodisperse nanoparticles were synthesized and subsequently validated by the presence of phosphate ester bonds in FTIR analysis and again the presence of the hydroxyapatite peak in the XRD analysis confirmed their successful formation. This study is the first demonstration of 5-FU encapsulation inside the core of inorganic calcium phosphate nanoparticles combined with efficient, in vitro cellular delivery. Furthermore, a sustained release profile and a high loading efficiency (64% drug loading into the nanoparticles) enabled improved, in vitro antineoplastic potential. Further, in vitro cell culture experiments (such as cell morphology studies like AO/EB staining and Hoechst staining) support the fact that apoptosis is one of the possible mechanisms through which cancer cells die. FE-SEM images of CaP@5-FU NP-treated cells revealed membrane bleb in both colorectal and lung cancer cell lines. Additionally, the increased intracellular ROS level confirmed the ROS inducing potential of CaP@5-FU NPs. Up-regulated proapoptotic genes (p53, caspase-3, BAX, BAK and BAD) and down-regulated antiapoptotic genes (Bcl-2 and Bcl-xL) found in the semiquantitative PCR analysis for gene expression studies further confirmed the apoptosis events during the CaP@5-FU NP treatment. Additionally, the intensified apoptotic expression in the cell population during the cell cycle analysis elucidated the role of CaP@5-FU NPs in apoptotic induction. Characteristic features such as high biocompatibility, bioavailability and pH-triggered behaviour, highlight the great potential of calcium phosphate nanoparticles as carriers for biomedical applications. Moreover, the low cost, highly reproducible microemulsion synthetic procedure further enhances the practicability of these materials for therapeutics. Given the results presented herein, we believe that the CaP@5-FU NP platform, providing increased efficacy in low dosage, will be an efficient means to replace some conventional chemotherapeutic strategies in the near future and to significantly reduce the mortality rate due to cancer.

## Experimental

### Materials

Calcium chloride and disodium hydrogen phosphate were procured from Himedia (Mumbai, India) and Merck (Germany), respectively. Dioctyl sulphosuccinate sodium salt (AOT) was purchased from Sigma-Aldrich (USA). Acridine orange (AO) and cellulose dialysis membrane-132 (molecular weight cut-off (MWCO) 12 kDa) were obtained from Himedia (India). Ethidium bromide (EtBr) was purchased from SRL (India). Fetal bovine serum (FBS) was bought from Gibco Scientific (USA). 3-(4,5-Dimethylthiazol-2-yl)-2,5-diphenyltetrazolium bromide (MTT) was obtained from Amresco Life Science (USA) and 3,8-diamino-5-[3(diethyl methyl ammonio)propyl]-6-phenylphenanthridinium diiodide (PI) from Sigma-Aldrich (USA). Superscript II reverse transcriptase, CellRox deep red, PCR supermix fluorescent probes, JC-9, random primers, dNTP mixture and RNase out were purchased from Invitrogen Company (California, USA). The primer pairs for PCR analysis to resolve apoptotic genes were obtained from Imperial Life Science (India). All other chemicals used in the present work were of analytical grade and were kept under the instructed storage conditions until used. All the cell lines were received from cell line repository (National Centre for Cell Science, Pune, India). All of the preparations were made in ultrapure water (18 MΩ·cm).

### Synthesis of 5-FU-incorporated CaP nanoparticles

5-FU-encapsulated CaP nanoparticles were prepared via a reverse micellar microemulsion chemical method. The synthesis procedure involved the use of the surfactant AOT in a nonpolar solvent. The synthesis procedure is schematically presented in Figure 1A. Briefly, in two separate parallel reaction systems, the precursor mixtures were stirred, where each was provided with the surfactant (0.1 M AOT) dissolved in n-hexane. The first reaction system (reverse micelle A) consisted of 0.1 M AOT, which self-assembled with 3.8 mM calcium chloride when the calcium precursor was added along with 200 µL of aqueous soluble 5-FU (10 mg/mL). The second reaction system (reverse micelle B) was comprised of 0.1 M AOT with 0.4 mM tris-HCl buffer (pH 7.4) and 1.05 mM Na_2_HPO_4_. A given amount of water was added to both reactions to provide a water–surfactant molar ratio (*M*_W_) of 10. Both of the reverse micelle systems were continuously stirred for about 12 h on a magnetic stirrer. Further, the first reverse micellar microemulsion system was mixed into the second set at an ultra-low speed of 0.1–1 mL/h and followed by stirring for up to 12 h under cold conditions (4 °C). The mixed reverse micellar microemulsions were optically translucent, confirming the formation of nanoparticles. This was followed by the addition of absolute ethanol, creating distinct aqueous and oil phases. The nanoparticles present in the aqueous phase were collected by centrifuging at 17,000 rpm for 20 min. Then the nanoparticles were washed three times with absolute ethanol to completely remove the surfactant from the aqueous phase. The pellet obtained was dispersed in PBS in a bath sonicator (30 Hz) for 5 min. Later, the aqueous dispersed solution was used for dialysis using a 12 kDa MWCO dialysis membrane and followed by lyophilization to yield nanoparticles in powder form. The powder samples were stored at −80 °C until used.

### 5-FU entrapment efficiency in CaP@5-FU NPs

The entrapment efficiency of 5-FU in CaP NPs was estimated by UV absorption studies. Initially, a calibration curve of 5-FU dissolved in PBS was determined by a Lasany double-beam L1 2800 UV–visible spectrometer. The standard curve (plotted at 265 nm, 0–50 μg/mL, *R* = 0.999) was used to calculate the amount of drug loaded into the NPs. A 5 mg sample of CaP@5-FU NPs were dispersed in 1 mL of PBS buffer and were sonicated with a pulse of 2 s “on” and 3 s “off” for about 15 min. The medium was further centrifuged at 17,000 rpm for 5 min to settle out the nanoparticle fragments. The entrapment efficiency (EE, wt %) of 5-FU in the CaP@5-FU NPs was calculated in the supernatant by the following equation:


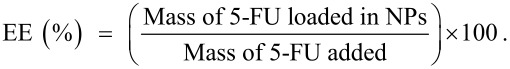


### UV–visible spectral characterization

The characterization of CaP@5-FU and CaP NP formation by UV–vis absorption spectroscopy was performed on a Lasany double-beam L1 2800 UV–visible spectrometer. The results were plotted using GraphPad Prism 6 software.

### pH-triggered in vitro release of 5-FU

The drug release profiles in the tumour microenvironment were evaluated using the dialysis bag method [53] in vitro model. Initially, 5 mg of CaP@5-FU NPs was dispersed in PBS and dialysed against two different pH environments, i.e., pH 5.5 in sodium acetate buffer and pH 7.4 in phosphate-buffered saline, under mild agitation. 1 mL of the dialysing media was periodically withdrawn (release medium) and the optical density of 5-FU at 265 nm was determined. The results, fit to a linear equation for 5-FU in release medium, were used for the estimation of the amount of drug released from the nanoparticles. The entire drug release study was carried out at 37 °C in order to mimic physiological conditions. The experiments were conducted in triplicate (*n* = 3) and the data presented as the mean ± SEM (standard error of mean).

### Hydrodynamic diameter and surface charge

The hydrodynamic diameter and surface charge of the CaP@5-FU and CaP NPs were measured in a Malvern Nano ZS 90 Zetasizer instrument at 25 °C. The diameter and zeta potential of 1 mg/mL CaP@5-FU NPs and CaP NPs in distilled water (pre-sonicated for homogenization) were measured in separate cuvettes.

### Fourier-transform infrared analysis

FTIR analysis was performed on both the CaP@5-FU NPs and CaP NPs to understand the chemistry of the functional group. The nanoparticle formation was confirmed by FTIR vibrational frequency analysis using a Thermo Nicolet spectrometer with the KBr pellet method in the range 4000–400 cm^−1^.

### X-ray diffraction studies

The crystalline properties of both CaP@5-FU and CaP NPs were explored by XRD analysis. 2 mg of powdered CaP@5-FU NPs and CaP NPs were analyzed in a Bruker AXS D8 advance X-ray diffractometer using Ni-filtered Cu Kα radiation with a typical scan speed of 0.05 °/min in the range of 2θ = 5–90°.

### Transmission electron microscopy

The surface and internal morphology as well as the size of CaP@5-FU NPs were visualised by TEM. A small drop of the aqueous dispersed CaP@5-FU NPs with the addition of 10 µL of 1% phosphotungstic acid was drop-cast onto a carbon-coated copper grid. This was followed by air drying of the wet grid under strict sink conditions. The grid was finally brought for examination in TEM instrument (FEI TECHNAI G2) operating at 200 keV.

### FE-SEM and AFM analysis

The characteristic surface morphology and size of the CaP@5-FU NPs was examined in a scanning electron microscope (Carl Zeiss ULTRA PLUS) operating at 5.0 kV and atomic force microscopy analysis (NTEGRA PNL) operating in semi-contact mode. An aqueous solution of CaP@5-FU NPs was dropped onto a glass slide and air dried. The particles were gold-sputtered for 60 s using a Denton gold sputter unit with vacuum conditions provided. The samples were then imaged under the FE-SEM and AFM instruments. The images were analysed with the help of ImageJ software to estimate the size distribution. The AFM images were further processed using NOVA RC1 software (version-1.0.26.922).

### Cell culture

The in vitro study was carried out in human colon cancer (HCT-15), lung adenocarcinoma (A549) and mouse fibroblast (NIH 3T3) cell lines and were procured from the National Centre for Cell Science, Pune, India. Human colon cancer (Duke’s type C, colorectal adenocarcinoma) HCT-15 cells were routinely maintained in Rosewell Park Memorial Institute 1640 (RPMI 1640) medium and subcultured on alternate days, whereas lung adenocarcinoma A549 and NIH 3T3 cells (mouse fibroblast cell line) were grown in Dulbecco’s modified Eagle’s medium (DMEM). Both cultures were provided with 10% fetal bovine serum (FBS) (Gibco Life Technologies, U.K.) and 1% penicillin-streptomycin (Sigma-Aldrich, USA). The cells were grown in a humidified incubator at a set temperature of 37 °C and 5% CO_2_ provided externally.

### Cell cytotoxicity test: MTT assay

The cytotoxic effect after a course of CaP@5-FU NPs, CaP NPs and free 5-FU treatment was determined by the mitochondrial dehydrogenase enzyme-mediated MTT assay. This included tests on preseeded HCT-15, A549 and NIH 3T3 cells at a density of 4 × 10^4^ cells per well in a 96-well cell culture plate and followed incubation for 24 h with varying concentrations of CaP@5-FU NPs, CaP NPs and free 5-FU. Then, the media was withdrawn to confirm the termination of nanoparticle incubation, and a PBS wash was performed followed by the addition of fresh media along with 10 µL MTT (5 mg/mL). After 3–4 hours, the formation of purple formazan was confirmed by microscopic observation and 100 µL DMSO was added to dissolve the formazan crystals. The formazan crystal formation was spectroscopically characterized using a multimode microplate reader (Cytation3, Biotek) at 570 nm absorbance. The percent of cell viability was calculated by a comparative study of the untreated control of the respective wells. The calculated cell viability was obtained by using the following equation. The data was presented as the mean ± SEM (*n* = 3).


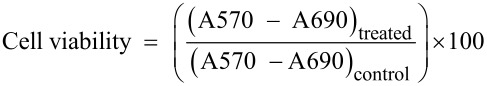


### Acridine orange/ethidium bromide (AO/EB) dual staining

Apoptotic induction after CaP@5-FU NP treatment was experimentally determined by dual staining assay (acridine orange/ethidium bromide (AO/EB)). Typically, AO/EB stained cells can distinguish the different phases of apoptosis upon incubation with an inducer [54]. The A549 and HCT-15 cells were seeded over a 35 mm dish at a seeding density of 3 × 10^5^ and followed incubation of IC_50_ concentration of CaP@5-FU NPs. The latter was removed along with the medium and the cells were stained with AO/EB (10 µg/mL) for 5–10 min after a PBS wash. After staining, a quick PBS wash was performed to remove free (unstained) AO/EB dye. Finally, images corresponding to the different phases of apoptosis were captured in an inverted phase contrast fluorescent microscope (EVOS cell imaging system) using corresponding fluorescent filters.

### Hoechst 33342 and rhodamine B dual staining

The combinatorial staining of rhodamine B and Hoechst 33342 were used as a tool to track the morphological changes occurring in the cells after treatment with CaP@5-FU NPs in a time-dependent manner. Hoechst 33342 can effectively stain the nucleic acid and specifically identify changes in the nucleus, whereas rhodamine B stains the cytoplasmic vesicles uniformly, enabling the cytoplasm to be visualised under a fluorescence microscope. The cells were seeded initially like the AO-EB assay, followed by incubation of an IC_50_ concentration of CaP@5-FU NPs for two time periods (i.e., 12 h and 24 h) along with an untreated control. This was followed by a PBS wash, and then the addition of 2 μL of 10 mg/mL Hoechst 33342 (incubated for 5 min) then observed under an EVOS fluorescence imaging system with a DAPI filter for the Hoechst 33342 blue fluorescence. This was followed by a PBS wash and addition of 3 µL of rhodamine B (1 mg/mL, incubated for 5–10 min) at 37 °C. Finally, images of the cells were captured by using blue (DAPI) and red (RFP) filters in the EVOS fluorescence imaging system and superimposed during image capturing.

### Membrane blebbing determination by FE-SEM

Once the understanding of apoptotic induction was confirmed by AO/EB staining, the membrane blebbing characteristic of apoptosis was determined upon CaP@5-FU NP treatment on both cancer cell lines (A549 and HCT-15) using FE-SEM. Both A549 and HCT-15 cells were seeded over an autoclaved glass coverslip in a 35 mm dish at a density of 3 × 10^5^. The seeded cells were grown overnight for proper attachment. This was followed by incubation at an IC_50_ concentration of CaP@5-FU NPs for around 24 h. The treated cells were later washed with PBS and processed with glutaraldehyde fixation along with a gradient (30%, 50%, 70%) ethanol fixation. In control cells, CaP@5-FU NPs were not added in order to compare the difference between treated and untreated cancer cells. When cells were treated with an IC_50_ value of CaP@5-FU NPs, they exhibited remarkable membrane blebbing. The cells on the coverslip were gold-sputtered using a Denton gold sputter unit and observed under FE-SEM (Ultra plus-Carl Zeiss) operating at 5 kV.

### Intracellular reactive oxide species quantification via flow cytometry

After the efficient cellular uptake of CaP@5-FU NPs, 5-FU was released into the cell. The introduction of 5-FU into the cell increases the ROS level and eventually leads to cell death. This was verified by quantification of the ROS-specific CellROX deep red fluorescence intensity through flow cytometry following the manufacturer’s protocol. The oxidative stress upon CaP@5-FU NP treatment in the HCT-15 cell line was characterized by this assay. Briefly, HCT-15 cells were grown in a 35 mm dish followed by treatment with an IC_50_ concentration of CaP@5-FU NPs for 24 h, followed by a PBS wash and trypsinization. Later, 5 µM of CellROX deep red was supplemented for the fluorescent staining followed by paraformaldehyde fixation. Finally, flow cytometry was performed on an Amnis Flowsight instrument (emdmillipore, USA) with all preliminary calibrations. CellROX deep red has an excitation and emission wavelength maxima at 644 nm and 665 nm, respectively. The data was acquired at 10,000 events per sample and post-processed by the Amnis Ideas software (version-6.1) provided by the manufacturer. The fluorescence intensity was measured with respect to the control.

### Cell cycle analysis

The different cell cycle phases were analysed using propidium iodide (PI) stained cells to evaluate the effect of the drug-loaded samples on the cellular metabolism and growth [47]. In brief, HCT-15 cells were incubated with an IC_50_ concentration of CaP@5-FU NPs and stained with PI. After a 24 h incubation time, the cells were wash with ice cold PBS and trypsinized and a cell pellet was obtained by centrifugation for 5 min at 4 °C. The cells were fixed with 70% ethanol and kept for 15 mins in ice cold conditions. Ribonuclease A (1 mg/mL^−1^ ) was added to exclude RNA interference in PI fluorescence. This was followed by fluorophore treatment with 0.1% Triton X-100 and PI (50–100 µg/mL). Finally, following the flow cytometry procedures as mentioned above, the cell cycles phases were determined by DNA quantification using PI fluorescence using the Amnis Ideas software.

### Semiquantitative RT-PCR assay for gene expression studies

The apoptotic induction was further verified by semiquantitative reverse transcription polymerase chain reaction (RT-PCR) on apoptotic and antiapoptotic genes. The cells were treated with their respective IC_50_ values of CaP@5-FU NPs and kept for 24 h under incubation. Later, total RNA was isolated from the cells by TRIzol reagent following the manufacturer’s protocol. From the total RNA, cDNA synthesis was performed with SuperScript II reverse transcriptase enzyme in a thermal cycler (Applied Biosystems). This was followed by gene-specific amplification using specific primer pairs for both apoptotic and antiapoptotic genes. The amplicons were qualitatively resolved in a 1.2% agarose gel electrophoresis under UV illumination of ethidium bromide. The untreated control cell’s gene expression was determined in a similar way for comparative analysis. The change in the gene expression was quantitatively determined by Image Lab 5.2.1 software by comparing with an untreated control gene expression. Both proapoptotic genes (p53, BAX, BAD, caspase-3) and antiapoptotic genes (Bcl-2 and Bcl-xL) were experimentally resolved. For internal control, β-actin (housekeeping gene) was utilized.

### Statistical analysis

The raw data were processed and plotted using GraphPad Prism 6 software (version 6.01). Statistical analysis was performed either by Student’s *t*-test or two-way ANOVA, as was applicable to the experiment. Tukey’s multiple comparisons test for statistical difference between the group means (*p < 0.05, **p < 0.005, ***p < 0.001, ns – non-significant) was implemented. The values are represented as the mean ± SEM (*n* = 3).

## Supporting Information

The supporting information contains an AFM image of CaP@5-FU NPs (Figure S1) and EDX data, the primer pairs of semiquantitative RT-PCR (Table S1), the MTT assay in the HEK 293 cell line (Figure S3), bright field images of HEK-293 and HCT-15 cells (Figure S4), and the mitochondrial membrane potential (Figure S5).

File 1 Additional experimental information.
