# Supplementary material for: Enhanced antineoplastic/therapeutic efficacy using 5-fluorouracil-loaded calcium phosphate nanoparticles
Source: Beilstein J Nanotechnol. 2018 Sep 20;9:2499–515. doi: 10.3762/bjnano.9.233 (PMC6176813; doi:10.3762/bjnano.9.233)
Supplement: File 1 — Additional experimental information. [file Beilstein_J_Nanotechnol-09-2499-s001.pdf]

Supporting Information

for

Enhanced antineoplastic/therapeutic efficacy using 5-fluorouracil-loaded calcium phosphate nanoparticles

Shanid Mohiyuddin[1†], Saba Naqvi[2†] and Gopinath Packirisamy[*1,2,§]

Address: [1]Department of Biotechnology, Indian Institute of Technology Roorkee, Roorkee, Uttarakhand-247667, India and [2]Nanobiotechnology Laboratory, Centre of Nanotechnology, Indian Institute of Technology Roorkee, Roorkee, Uttarakhand-247667, India

Email: Gopinath Packirisamy - genegopi@gmail.com

*****Corresponding author

[†]These authors contributed equally to the work

[§]Fax: +91-1332-273560; Tel: 91-1332-285650

Additional experimental information

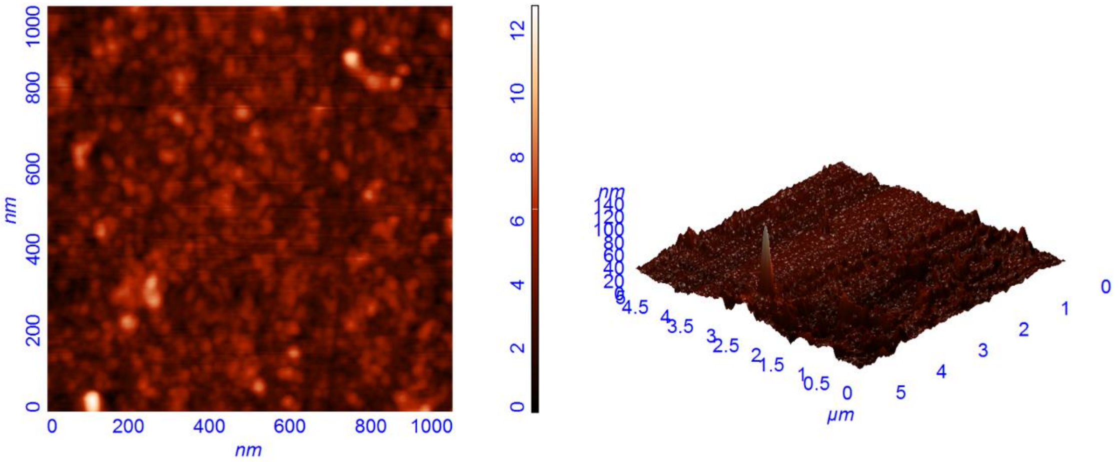

Figure S1: Atomic force microscopic image of CaP@5-FU Np’s.

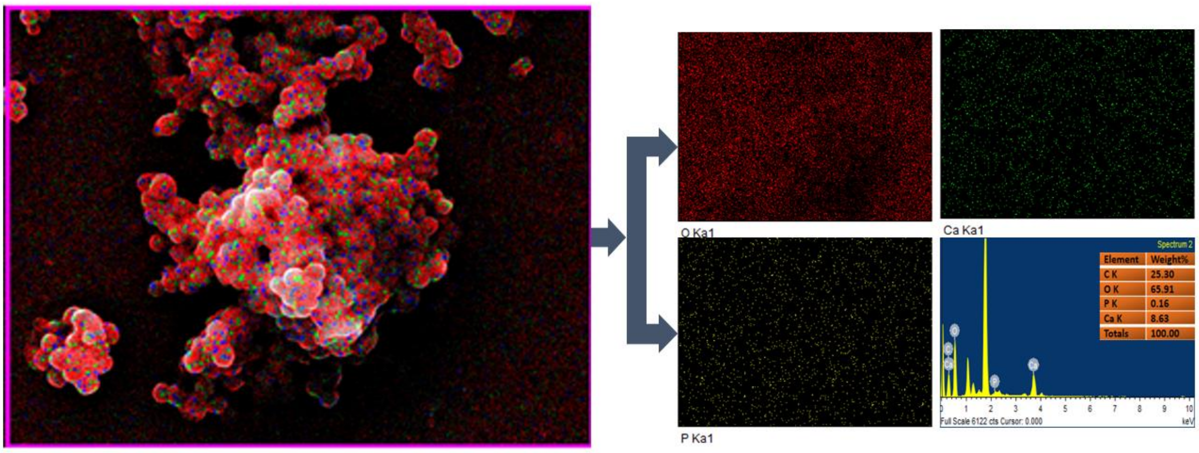

Figure S2: EDX analysis for elemental determination via FE-SEM of CaP@5-FU Np’s with weight percentage as assessed.

Table S1: The primer pair for pro-apoptotic (Bax, Bad, Bak, p^{53} and Caspase) and anti-apoptotic (Bcl-2 and Bcl-xL) genes used in semi-quantitative Reverse Transcriptase PCR.

β-actin	Forward: 5′-CTGTCTGGCGGCACCACCAT-3′<br>Reverse: 5′-GCAACTAAGTCATAGTCCGC-3′
Bcl-2	Forward: 5′-AGATGTCCAGCCAGCTGCACCTGAC-3′<br>Reverse: 5′-AGATAGGCACCCAGGGTGATGCAAGCT-3′
Caspase-3	Forward: 5′-TTTGTTTGTGTGCTTCTGAGCC-3′<br>Reverse: 5′-ATTCTGTTGCCACCTTTCGG-3′
Bak	Forward: 5′-TCCAGATGCCGGGAATGCACTGACG-3′<br>Reverse: 5′-TGGTGGGAATGGGCTCTCACAAGG-3′
Bcl X_L	Forward: 5′-ATGGCAGCAGTAAAGCAAGCGC-3′<br>Reverse: 5′-TTCTCCTGGTGGCAATGGCG-3′
p53	Forward: 5′-TGGCCCCTCCTCAGCATCTTAT-3′<br>Reverse: 5′-GTTGGGCAGTGCTCGCTTAGTG-3′
Bad	Forward: 5′-CCTTTAAGAAGGGACTTCCTCGCC-3′<br>Reverse: 5′-ACTTCCGATGGGACCAAGCCTTCC-3′
Bax	Forward: 5′-AAGCTGAGCGAGTGTCTCAAGCGC-3′<br>Reverse: 5′-TCCCGCCACAAAGATGGTCACG-3′

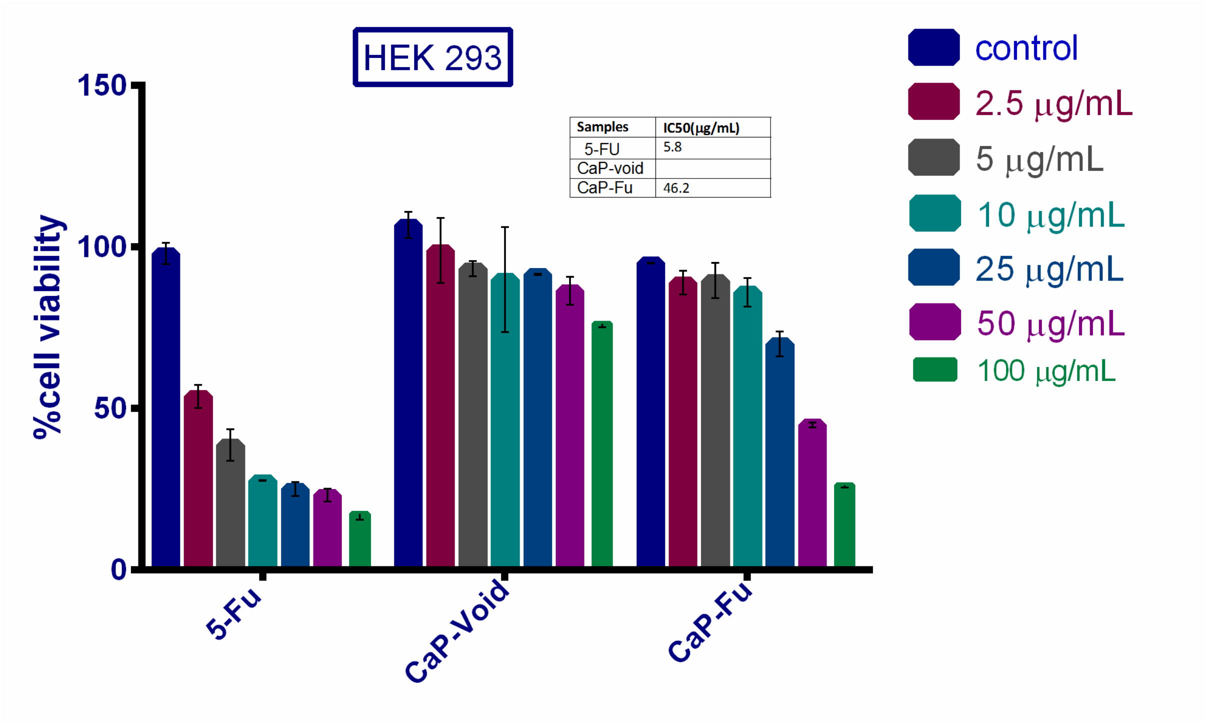

Figure S3: MTT assay of CaP@5-FU Np's, CaP Np's and 5-FU in HEK 293 cell line.

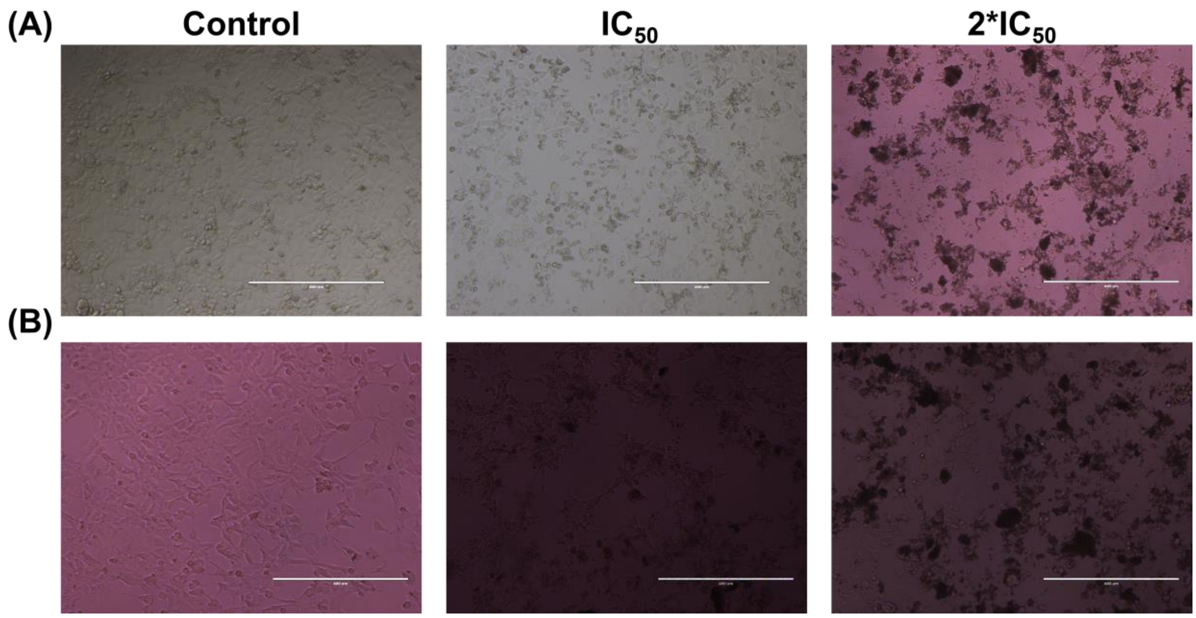

Figure S4: Bright field morphological images of (A) HCT-15 and (B) HEK-293 cells upon treatment of CaP@5-FU with IC_{50} and 2* IC_{50} concentration.

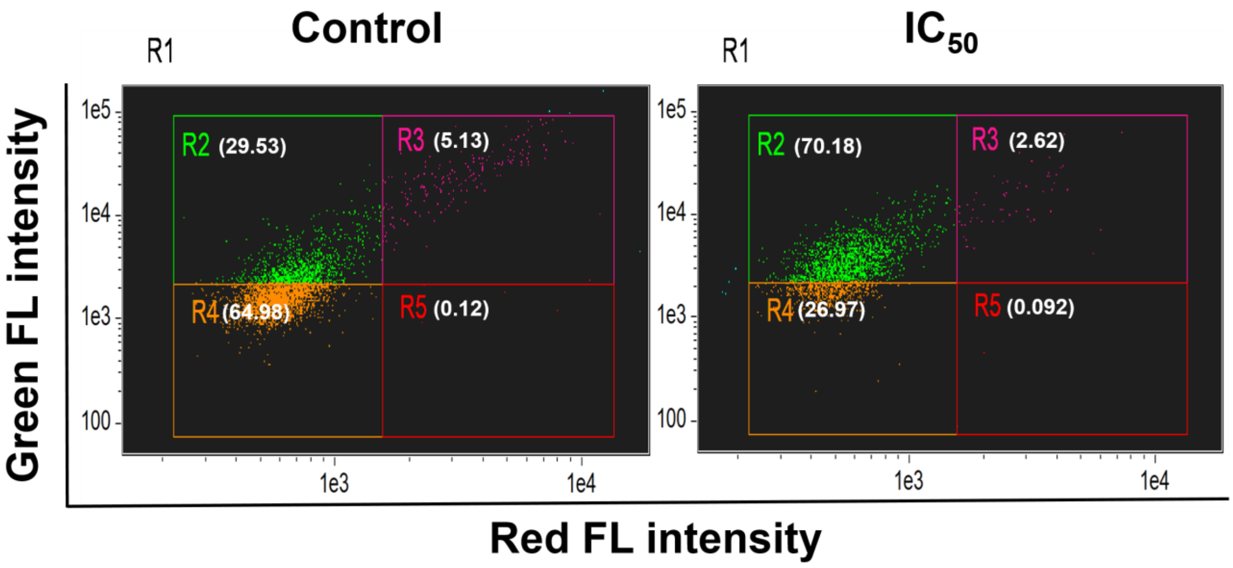

Figure S5: Flow cytometric data of JC-9 fluorescence quantification of CaP@5-FU with IC_{50} treated HCT-15 cells for mitochondrial membrane potential.
